# Surface Deposition and Coalescence and Coacervation Phase Separation Methods:* In Vitro* Study and Compatibility Analysis of Eudragit RS30D, Eudragit RL30D, and Carbopol-PLA Loaded Metronidazole Microspheres

**DOI:** 10.1155/2015/254930

**Published:** 2015-11-16

**Authors:** Irin Dewan, Md. Maynul Islam, Maksud Al-Hasan, Joydeb Nath, Sefat Sultana, Md. Sohel Rana

**Affiliations:** ^1^Department of Pharmacy, University of Asia Pacific, Dhanmondi, Dhaka 1209, Bangladesh; ^2^Department of Pharmacy, Jahangirnagar University, Savar, Dhaka 1342, Bangladesh

## Abstract

Metronidazole (MTZ) has extremely broad spectrum of protozoal and antimicrobial activity and is clinically effective in trichomoniasis, amoebic colitis, and giardiasis. This study was performed to formulate and evaluate the MTZ loaded microspheres by coacervation phase separation and surface deposition and coalescence methods using different polymers like Gelatin, Carbopol 934P, Polylactic Acid (PLA), Eudragit RS30D, and Eudragit RL30D to acquire sustained release of drug.* In vitro* dissolution studies were carried out in phosphate buffer (pH 7.4) for 8 hours according to USP paddle method. The maximum and minimum release of MTZ from microspheres observed were 84.81% and 76.6% for coacervation and 95.07% and 80.07% for surface deposition method, respectively, after 8 hours. Release kinetics was studied in different mathematical release models. The SEM and FTIR studies confirm good spheres and smooth surface as well as interaction between drug and polymers. Though release kinetic is uncertain, the best fit was obtained with the Korsmeyer kinetic model with release exponent (*n*) lying between 0.45 and 0.89.* In vitro* studies showed that MTZ microspheres with different polymers might be a good candidate as sustained drug delivery system to treat bacterial infections.

## 1. Introduction

In the early 1950s Barrett K. Green developed the microencapsulation that used the process of coacervation. The first pharmaceutical product consisting of microcapsules was a controlled release aspirin product. The oral route is considered as the most promising route of drug delivery. Conventional drug delivery system achieves and maintains the drug concentration within the therapeutically effective range needed for treatment, only when taken several times a day. This results in a significant fluctuation in drug levels. A well-defined controlled drug delivery system can overcome some of the problems of conventional therapy and enhance the therapeutic efficacy of a given drug [1]. For colonic drug delivery, many physiological barriers must be overcome, the major one being absorption or degradation of the active drugs in the upper part of the GIT. Most of the peptide and protein drugs are unstable in the stomach and upper part of the intestine. Colon specific drug delivery protects peptide drug from releasing. After bioavailability, there should be no space before the period. To achieve successful colonic delivery, a drug needs to be protected from absorption and/or degradation in the environment of the upper GIT [2].

A variety of controlled release systems such as coated pellets, matrix tablets, osmotically controlled release systems, microcapsules, microspheres, nanoparticles, implants, and infusion devices have been designed for various routes of drug administration. Controlled release formulations in tablet form are many, but over the years the microsphere formulations have immense popularity owing to their superiority over the former in several respects [3]. A multiple-unit dosage form has more homogenous individual plasma profiles, shorter lag time, and lower variability as compared to single-unit formulations. The uniform distribution of these multiple unit dosage forms along the GIT could result in more reproducible drug absorption and reduced risk of local irritations than the use of single-unit dosage forms. Risks such as spontaneous drug release from a single-unit tablet due to damaged coating or its attachment in the stomach or intestine causing an irritation of the gastric or intestinal mucosa are reduced by the use of multiunit forms. Thus, it results in a decrease in drug dose and side effects [4].

Due to the smallness of the particles, drugs can be widely distributed throughout the GI tract, hence improving the drug absorption. The microencapsulation technique offers a variety of opportunities such as protection and masking, reduced dissolution rate, facilitation of handling, and spatial targeting of the active ingredient. This approach facilitates accurate delivery of small quantities of potent drugs, reduced drug concentrations at sites other than the target organ or tissue, and protection of labile compounds before and after administration and prior to appearance at the site of action. In the future by combining various other approaches, microencapsulation technique will find the vital place in novel drug delivery system [5].

Metronidazole, a nitroimidazole, has extremely broad spectrum of antiprotozoal and antimicrobial activities, with high activity against anaerobic bacteria. Metronidazole is sparingly soluble in water and alcohol and slightly soluble in ether, chloroform, acetone, and methanol. Half-life of metronidazole is about 6 to 7 hours [6]. The sustained release MTZ microspheres can be prepared by different microencapsulation technique. Microencapsulation is the process in which small droplets or particles of liquid or solid material are surrounded or coated by a continuous film of polymeric materials. The purpose of microencapsulation is to improve the solubility, increase bioavailability, and delay the release of drug [7]. Coacervation phase separation process may be used to microencapsulate a variety of liquids, solids, solutions, and dispersions of solids in liquids. The polymers used to coat the materials should be soluble in water or any other solvent used. Water-soluble core materials are microencapsulated in organic solvents, whereas water-insoluble materials are microencapsulated in water [8].

Methacrylate copolymers (Eudragits) have recently received increased attention for modified dosage forms because of their inertness, solubility in relatively nontoxic solvents, and availability of resins with different properties. Eudragit RS30D and Eudragit RL30D are a copolymer of ethyl acrylate, methyl methacrylate, and a low content of methacrylic acid ester with quaternary ammonium groups. The ammonium groups are present as salts and make the polymers permeable [9]. Carbopol is a polymer consisting of acrylic acid cross-linked with either polyalkenyl ether or divinyl glycol. It readily absorbs water, gets hydrated, and swells. In addition to its hydrophilic nature, cross-linked structure, and insolubility in water, Carbopol is an anionic polymer and, therefore, a potential candidate for use in controlled release drug delivery [10]. Polymers such as polylactide (PLA) and poly(lactide-co-glycolide) (PLGA) have been successfully used to prepare micro- and nanoparticulate drug delivery systems as well as other medical devices, like sutures and implants. Pharmaceutical products based on PLA and PLGA microparticles loaded with hormones, antitumor drugs, and antibiotics are already available in the market in Europe and the US [11].

The aim of the present investigation was to formulate and* in vitro* evaluate colon targeted microspheres of metronidazole using a combination of time controlled and pH independent polymethacrylate polymers that offer protection to the drug until it leaves the stomach and major drug release in small intestine is avoided by providing pH independent coating of Eudragits RS and RL.

## 2. Materials and Methods

### 2.1. Materials

The materials used were metronidazole as a donation sample from SQUARE Pharmaceuticals Ltd., Bangladesh, Carbopol 934P (Colorcon Asia Pvt. Limited, India), Polylactic Acid (PLA) (Merck, Germany), Eudragit RS30D (Evonik, Germany), Eudragit RL30D (Evonik, Germany), light liquid paraffin, petroleum ether (Merck, Germany), sodium hydroxide (Merck, Germany), potassium dihydrogen phosphate (Merck, Germany), and distilled water.

### 2.2. Methods

#### 2.2.1. Preparation of Metronidazole Microspheres by Coacervation Phase Separation Method

**Figure 1:**
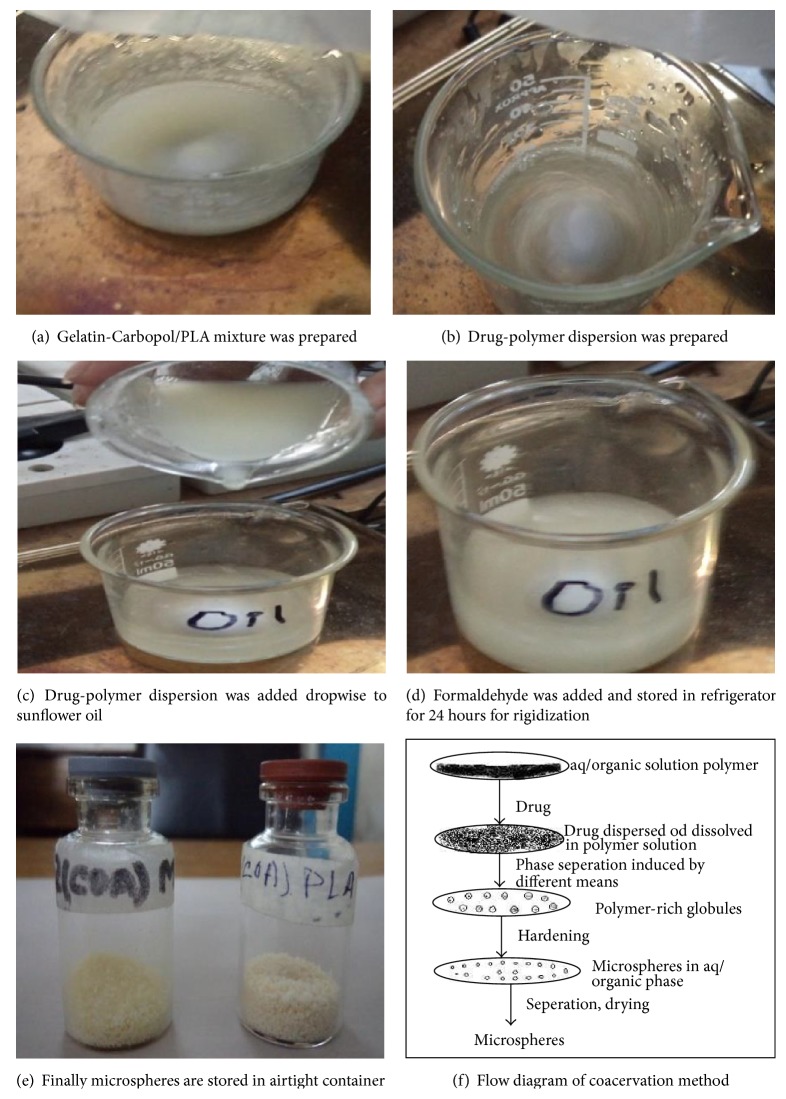
It schematically presents the steps of microspheres prepared by coacervation phase separation method.

According to Table 1 Gelatin-Carbopol/PLA mixture was dissolved in ten mL of water which was previously heated to 50°C. To this metronidazole was added and stirred approximately at 300 rpm with the help of magnetic stirrer for 15 minutes to get a stable dispersion. The dispersion was poured dropwise into the ten mL of sunflower oil which was also previously heated to 50°C on a water bath. The mixture was stirred with a help of magnetic stirrer for 2 hrs at 300 rpm at room temperature. At the end of two hrs, cross-linking agent formaldehyde 0.5 mL was added to the dispersion medium with continuous stirring for next 30 minutes. After that, the final dispersion was kept in refrigerator for 24 hrs to make sure of the rigidization of microspheres shown in Figure 1.

#### 2.2.2. Preparation of Metronidazole Microspheres Based on Surface Deposition and Coalescence Method

**Step 1 (preparation of granules using Eudragit RL/RS30D by wet granulation method).**

For the preparation of granules at first ten grams (10 gm) of drug was placed in a beaker and 6.6 gm of Eudragit RL/RS30D was added to it in aliquots with proper mixing. The wet mass was air-dried and passed twice through 30-mesh sieve and kept in an oven at 40°C for 2 hours. After drying the granules were sieved through 710-, 500-, and 250-micron-size sieves and collected in different size ranges.

**Step 2 (preparation of microspheres using emulsion solvent evaporation method).**

According to Table 1 about 300 gm of light liquid paraffin oil and 1 gm of span 20 were mixed in a beaker with continuous stirring at 2000 rpm for few minutes. The whole mixture was divided into two parts (part A: 200 gm and part B: 100 gm).

**Figure 2:**
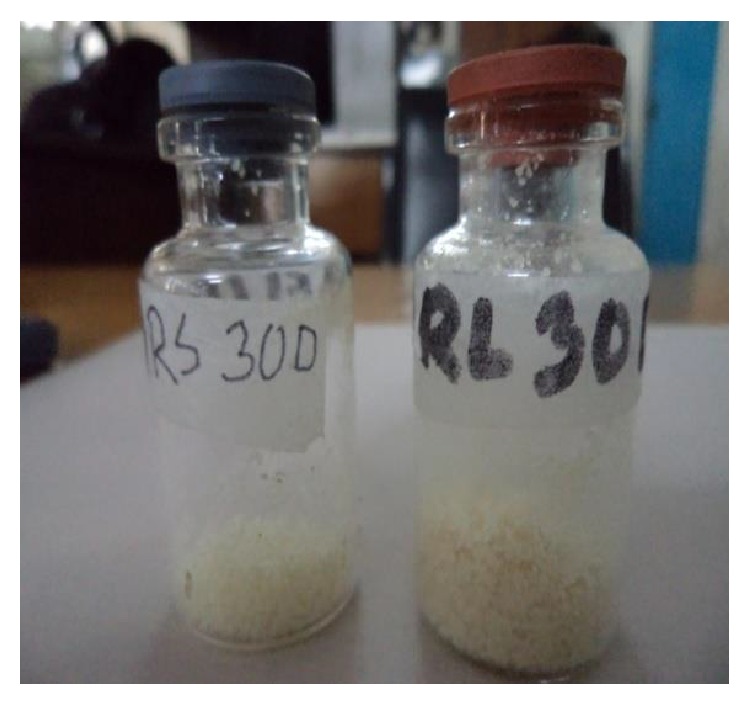
Formulation of metronidazole microspheres based on surface deposition and coalescence method.

Part A was taken in a beaker and an emulsion was made with 18 gm Eudragit RL/RS30D. Part B was taken in another beaker and placed onto a hot plate. The temperature was raised to 90°C. About 4 gm of 250–500-micron-sized metronidazole granules was added to this part of light liquid paraffin with continuous stirring. Part A containing Eudragit RL/RS30D emulsion was added in different installment. Few-minute time was allowed between each addition to settle down the Eudragit RL/RS30D over the granules. The temperature was maintained at 85 to 90°C for 90 minutes. After cooling to room temperature the microspheres were filtered and washed three times with petroleum ether and those were kept in dry air. Finally the microspheres were dried at 40°C for 12 hours shown in Figure 2.

### 2.3. Assay Methods of Prepared Microspheres

Approximately 20 mg of microspheres was taken in a 100 mL volumetric flask and dissolved in up to phosphate buffer (pH 7.4) and thoroughly shaken in ultrasonic bath at 37°C for 30 minutes. Then the solution was filtered through Whatman filter paper and analyzed spectrophotometrically at 277 nm for the drug concentration. The drug loading was calculated by using the following equation:

(1) %Drug Loading=Actual Drug LoadingMicrospheres to Be Taken×100%.

The drug entrapment efficiency was calculated by using the following equation:

(2) %Drug Entrapment Efficiency=Actual Drug ContentTheoretical Drug Content×100%.

### 2.4. *In Vitro* Release Study of Metronidazole Microspheres

*In vitro* dissolution study was performed in a paddle type dissolution apparatus. Nine hundred milliliters (900 mL) of phosphate buffer (pH 7.4) was used as dissolution media, paddle speed was 100 rpm, and temperature was maintained fixed at 37°C. Approximately 20 mg of microspheres from each batch was transferred into each dissolution basket. The dissolution process was carried out for 8 hours and 10 mL dissolution sample was withdrawn at predetermined different time intervals and replaced with the same volume of test medium to maintain sink conditions. Dissolution samples were withdrawn with the help of 10 mL syringe and kept in test tubes. The withdrawn samples were diluted, where necessary, filtered through 0.45 *μ* membrane filter, and analyzed in UV-VIS spectrophotometer at a wavelength of 277 nm.

### 2.5. Kinetic Analysis of Dissolution Data

To study the mechanism of drug release from the microspheres, the release data were fitted to zero order, first order, and Higuchi equation [12]. Moreover, for better characterization of the drug release mechanisms, the Korsmeyer-Peppas [13] semiempirical model was applied:

(3) $$\begin{array}{c} \frac{Q_{t}}{Q_{e}}=k_{\text{KP}}t^{n}, \end{array}$$

where *Q*_*t*_/*Q*_*e*_ is the fraction of the drug released at time *t*, *k*_KP_ is a constant corresponding to the structural and geometric characteristics of the device, and *n* is the release exponent which is indicative of the mechanism of the drug release. To clarify the release exponent batches of microspheres, the log value of percentage drug dissolved was plotted against log time for each batch according to the above equation. A value of *n* = 0.45 indicates Fickian (case I) release; >0.45 but <0.89 indicates non-Fickian (anomalous) release; and >0.89 indicates super case II type of release. Case II generally refers to the erosion of the polymeric chain and anomalous transport (non-Fickian) refers to a combination of both diffusion and erosion controlled drug release [14].

### 2.6. Surface Morphology Study with the Help of Scanning Electron Microscope (SEM)

Surface nature of microspheres was examined with the help of Scanning Electron Microscope (JEOL, JSM-6490 LA, Japan). The microspheres were dried completely before examination. SEM was done at different magnifications of 20.0 kv ×75, 20.0 kv ×90, 20.0 kv ×95, 20.0 kv ×140, 20.0 kv ×300, 20.0 kv ×600, and 20.0 kv ×1000.

### 2.7. Fourier Transform Infrared (FTIR) Spectroscopy Studies

The FTIR technique is to measure the absorption of various infrared radiations by the target material and to produce an IR spectrum that can be used to identify functional groups and molecular structure in the sample shown in Figure 7. FTIR spectra of pure MTZ and formulated microspheres were recorded by using FTIR 8400S (SHIMADZU, Japan). Appropriate quantities of KBr and microspheres (in the ratio 100 : 2) were mixed by grinding in an agate mortar. Disk was made with about 100 mg mixture under hydraulic pressure of 600 kg. Then the FTIR spectra were recorded between 4000 and 400 cm^−1^. The resolution was 2 cm^−1^.

## 3. Results and Discussions

### 3.1. Actual Drug Loaded and Drug Entrapment Efficiency (DEE) of Prepared Microspheres

**Figure 3:**
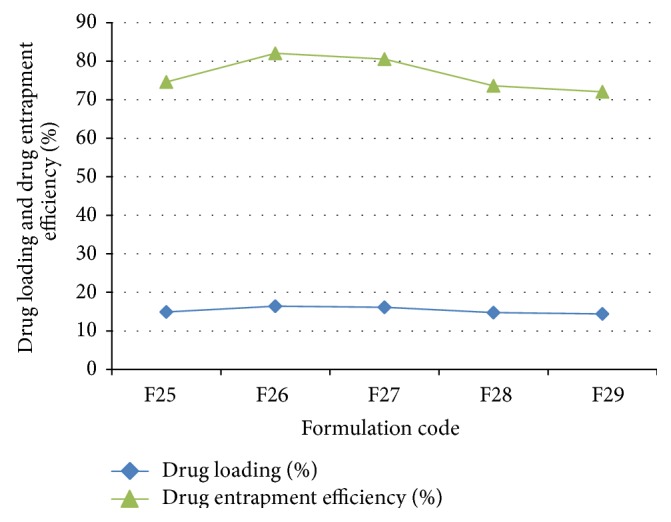
Comparative percent release study of actual drug loading and drug entrapment efficiency of different formulations.

Drug loading and the drug entrapment efficiency (DEE) of the prepared microspheres were carried out and the graphical presentation is given in Figure 3. The actual drug loaded and the drug entrapment efficiency were found to be in the range of 14.48% to 16.48% and 72.04% to 82.04%, respectively.

### 3.2. Dissolution Study of Metronidazole Microspheres Prepared by Coacervation Phase and Surface Deposition and Coalescence Methods

To find out the mechanism of drug release, the controlled release MTZ microspheres were treated in different mathematical models like zero order (cumulative percentage of drug release versus time), first order (log percentage of drug remaining versus time), Higuchi model (cumulative percentage of drug release versus square root of time), and Korsmeyer model (log cumulative percentage of drug release versus log time). The release data was plotted. From the linear portions of the curve slope correlation coefficients (*R* ^2^) were calculated. With the Korsmeyer plot, linearity was noted highest in all formulations using all data points. The data yielded apparently straight line with Korsmeyer plot (*R* ^2^ > 0.99) while a bit with zero order, first order kinetics and Higuchi plot. It is observed that drug released from sustained release microsphere followed Korsmeyer release log cumulative percentage of drug release versus log time. The mechanism of drug release was calculated according to Peppas equation. The calculated “*n*” values along with the correlation coefficients (*R* ^2^) have been shown in Table 2. The values of *n* depend upon the polymer concentration. The calculated “*n*” values suggest that the mechanism of drug release followed non-Fickian transport.

### 3.3. Effect of Polymers (Gelatin, Carbopol, and Polylactic Acid) and (Eudragit RL30D and Eudragit RS30D) on the Release of Metronidazole Microspheres Prepared by Coacervation Phase Separation and Surface Deposition and Coalescence Methods, Respectively

Metronidazole microspheres were prepared by polymeric concentration variation to study the effect of combination polymer on the release of drug from microspheres.

**Figure 4:**
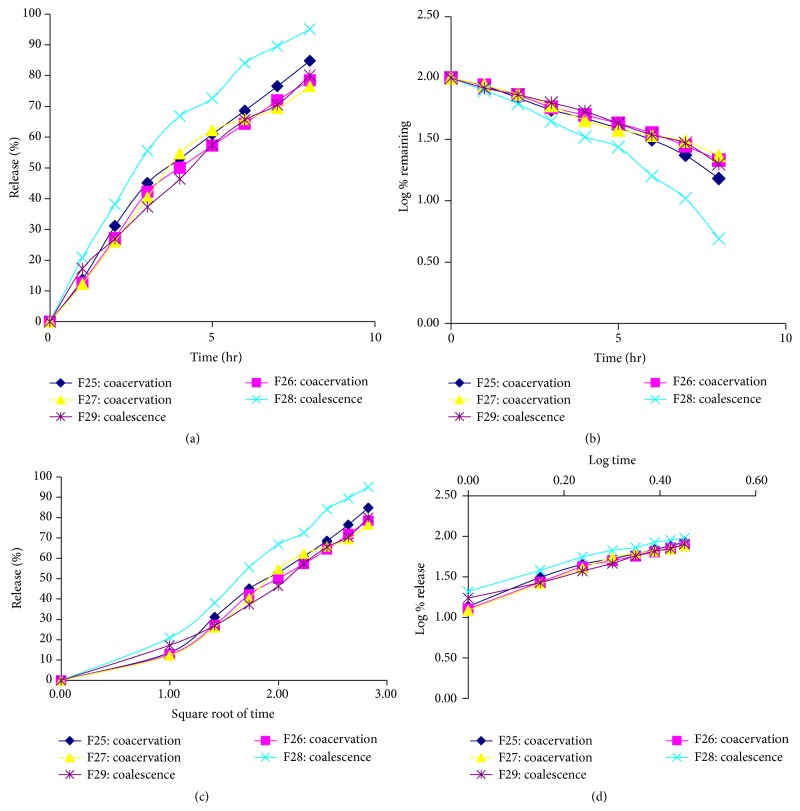
Release of metronidazole from formulations F25 to F27 and formulations F28 to F29 by coacervation phase separation and surface deposition and coalescence method, respectively. (a) Zero order, (b) first order, and (c) Higuchi and (d) Korsmeyer models.

Polymers such as Gelatin, Carbopol, and Polylactic Acid (PLA) in different concentrations were used in formulations F25 to F27. The initial burst release of formulations F25, F26, and F27 was about 13.68%, 12.73%, and 12.26%, respectively, after 1 hour (Figure 4). After the end of 8 hours of dissolution, the release of microspheres from F25, F26, and F27 was 84.81%, 78.49%, and 76.60%, respectively. The addition of Carbopol along with Gelatin in formulation F26 retards the rate of drug release. The addition of PLA along with the same concentration of Gelatin in formulation F27 retards the rate of drug release significantly. Besides formulations F28 and F29 were prepared using the polymers of Eudragit RL30D and Eudragit RS30D in different concentrations. The initial burst release of formulations F28 and F29 was about 20.94% and 17.31%, respectively, after 1 hour (Figure 4). After the end of 8 hours of dissolution, the release of microspheres from F28 and F29 was 95.07% and 80.07%, respectively. The release of drug is significantly higher for formulation F28 than formulation F29. Eudragit RS30D retards the release of drug more than Eudragit RL30D.

All the formulations were best fitted with Korsmeyer model as shown in Table 2. The release exponent *n* > 0.45 and *n* < 0.89 for all the formulations showed non-Fickian (case II) anomalous release of drug which refers to a combination of both diffusion and erosion controlled drug release.

### 3.4. Effect of Polymers Concentration on the Surface Morphology of Metronidazole Microspheres Prepared by Coacervation Phase Separation Method

**Figure 5:**
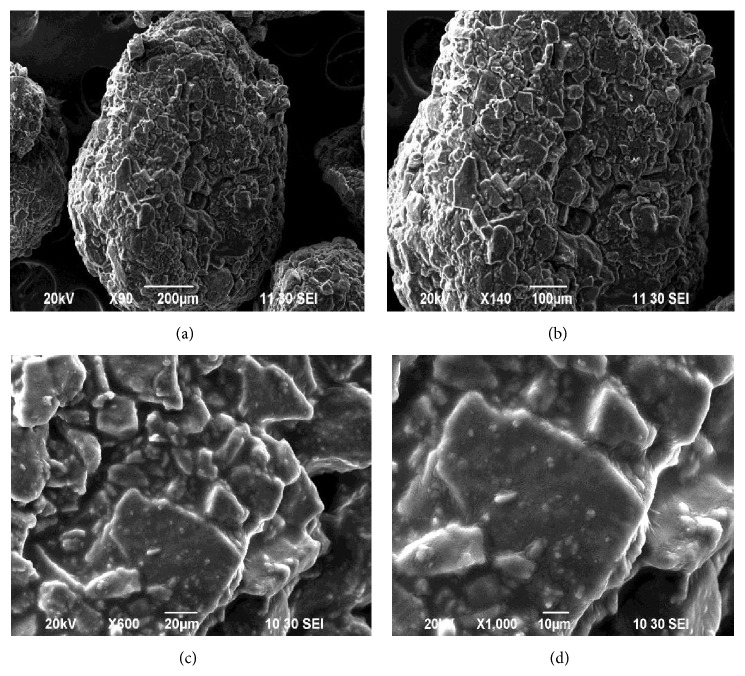
SEM studies of metronidazole microspheres of formulation F25 prepared by coacervation phase separation method with different magnification: (a) at ×90 SEI, (b) at ×140 SEI, (c) at ×600 SEI, and (d) at ×1000 SEI.

SEM study has shown that microspheres of formulation F25 were spherical and aggregated. The surface of the drug loaded microspheres manifested the presence of drug particles and rough surface, clearly visible from outside at high magnification in Figure 5.

### 3.5. Effect of Polymers Concentration on the Surface Morphology of Metronidazole Microspheres Prepared by Surface Deposition and Coalescence Method

**Figure 6:**
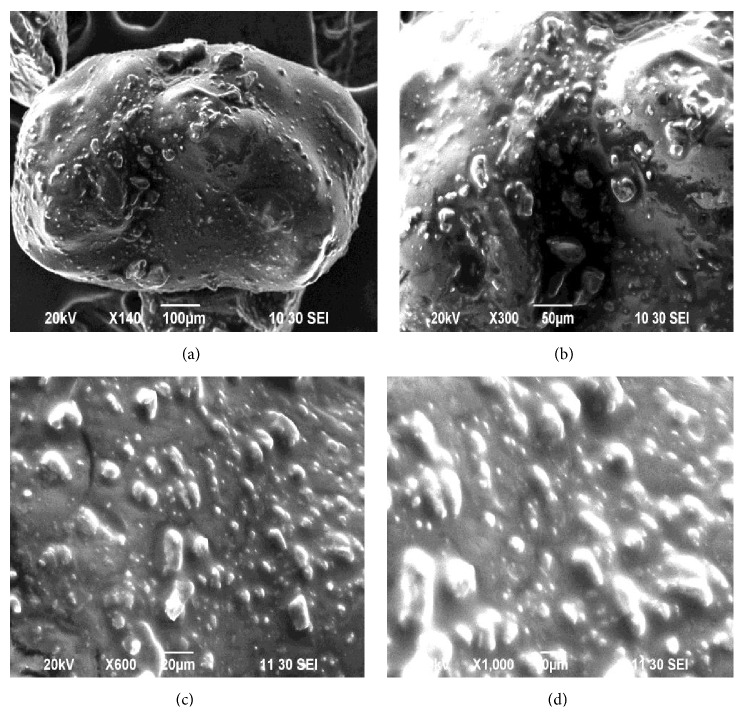
SEM studies of metronidazole microspheres of formulation F28 prepared by surface deposition and coalescence method with different magnification: (a) at ×140 SEI, (b) at ×300 SEI, (c) at ×600 SEI, and (d) at ×1000 SEI.

Formulation F28 has contained the polymer Eudragit RL30D. The microspheres were found in spherical shape and surface was smooth as shown in Figure 6. The magnification of microcapsules showed that the polymers surround the drug.

### 3.6. Drug-Polymer Compatibility Study by Fourier Transform Infrared (FTIR) Spectroscopy

**Figure 7:**
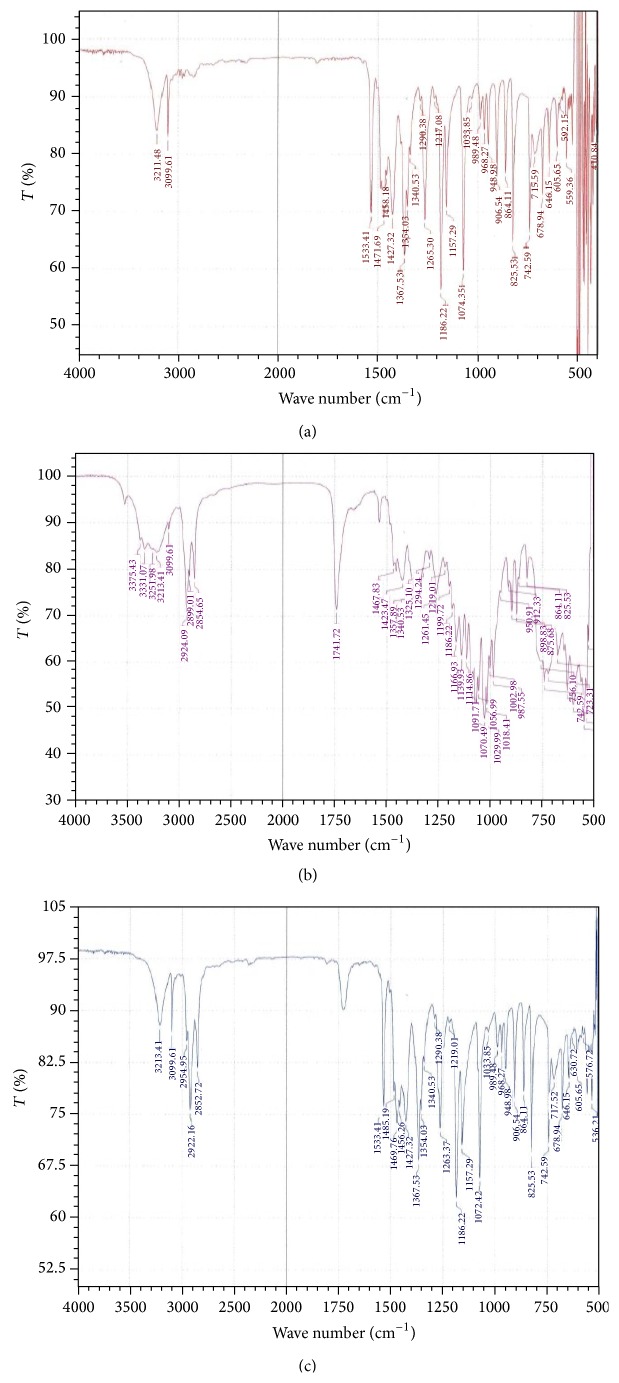
FTIR spectrum of (a) pure drug metronidazole, (b) formulation F26, (c) formulation F28, Eudragit RS30D, and Eudragit RL30D, respectively. %  *T*: % transmittance or absorbance.

FTIR spectra of pure MTZ and its combination with polymers were shown in Figure 7. An FTIR spectrum of pure metronidazole showed the peaks such as O–H stretch (3211.8 cm^−1^), C=CH, C–H stretch (3099.61 cm^−1^), NO2, N–O stretch (1533.41 cm^−1^), C–OH, C–O bend (1074.35 cm^−1^), and C–NO2, C–N stretch (825.53 cm^−1^) that are shown in Table 3. These peaks can be considered as characteristic peaks of drug and were not affected and prominently observed in IR spectra of pure drug along with polymers as shown in Figures 7(b) and 7(c), indicating no interaction between drug and polymers. These peaks indicated that MTZ is entrapped here. As various polymers were used in those formulations in different amounts, the IR spectra were different from the active one.

## 4. Conclusion

The present study was conducted to design metronidazole sustained release microspheres by coacervation phase separation and surface deposition and coalescence method. Different polymers of different concentrations were used in the preparations to observe the surface of microspheres and thus the release characteristics of drug. As the concentration of polymer increases, the release rate decreases gradually and the release studies showed that highest concentration of polymer gave the best sustained effect. All the formulations were best fitted with Korsmeyer model. The release exponent *n* < 0.89 for all the formulations showed non-Fickian (case II) anomalous release of drug which refers to a combination of both diffusion and erosion controlled drug release.

Further experiment should be performed to establish more sustained effect. This approach facilitates accurate delivery of small quantities of potent drugs, reduced drug concentrations at sites other than the target organ or tissue, and protection of labile compounds before and after administration and prior to appearance at the site of action.* In vitro* studies showed that MTZ microspheres with different polymers might be a good candidate as sustained drug delivery system to treat bacterial infections. It can be concluded that polymers lower the release rate of the drug and may prolong the activity and overall release kinetics.

## Figures and Tables

**Table 1:** Formulation and drug loading efficiency of metronidazole microspheres prepared by coacervation phase separation method and surface deposition and coalescence.

Formulation code	Drug (gm)	Polymers (gm)			Formaldehyde (mL)	AL (%)	DEE (%)	Methods
		Gela	Car	PLA				
F25	1	1	0	0	0.5	14.91	74.58	*Coacervation phase separation*
F26	1	1	0.5	0	0.5	16.48	82.04	
F27	1	1	0	0.5	0.5	16.11	80.55	
F28	10	EuRS30D: 6.6	—	—	—	14.72	73.62	*Surface deposition and coalescence*
F29	10	EuRL30D: 6.6	—	—	—	14.48	72.04	

Gela: Gelatin, Car: Carbopol, PLA: Polylactic Acid, EuRS30D: Eudragit RS30 dispersion, EuRL30D: Eudragit RL30 dispersion, DEE: drug entrapment efficiency, AL: actual loading, mL: milliliter, and gm: gram.

**Table 2:** Correlation coefficient (*R* ^2^) of different formulations of metronidazole microspheres using different polymers by coacervation and coalescence methods, respectively.

Formulation code	Zero order	First order	Higuchi model	Korsmeyer model		
	*R* ^2^	*R* ^2^	*R* ^2^	*R* ^2^	*K*	*n*
F25	0.9721	0.9773	0.9682	0.9795	15.71	0.8405
F26	0.9716	0.9805	0.9659	0.9935	14.32	0.8543
F27	0.9483	0.9756	0.9573	0.9891	13.88	0.879
F28	0.9494	0.9633	0.9809	0.984	22.70	0.7253
F29	0.9855	0.9811	0.9642	0.9971	16.72	0.7488

**Table 3:** Functional group detection using wave number.

Functional group	Wave number in 1/cm		
	Range	Formulation F26	Formulation F28
C-H alkenes	700–610	677.01–630.72	678.94–630.72
C-CI	735–702	723.31	717.52
C-N (bend)	1000–750	987.55–756.1	989.48–825.53
C-O (bend)	1300–1000	1294.24–1002.98	1290.38 –1033.85
C-N	1350–1000	1340.53–1002.98	1340.53–1033.85
N=O	1550–1350	1467.83–1357.89	1533.41–1354.03
-CHO	1720–1740		
Aliphatic C-H (stretch)	3100–2850	3099.61–2854.65	3099.61–2852
O-H	3400–3200	3375.43–3213.41	3213.41
